# Time to Death and Its Determinant Factors Among Patients With Chronic Heart Failure in Northwest Ethiopia: A Retrospective Study at Selected Referral Hospitals

**DOI:** 10.3389/fcvm.2022.817074

**Published:** 2022-05-06

**Authors:** Yikeber Abebaw Moyehodie, Mitiku Wale Muluneh, Alebachew Taye Belay, Setegn Muche Fenta

**Affiliations:** Department of Statistics, Debre Tabor University, Debre Tabor, Ethiopia

**Keywords:** Cox, heart failure, hospital, multicenter, mortality, survival

## Abstract

**Background:**

Heart failure (HF) is a major health problem that affects patients and healthcare systems worldwide. It is the leading cause of morbidity and death and negatively impacts the quality of life, healthcare costs, and longevity. However, the causes of death were not well defined. This study aimed to identify the determinants of death among patients with HF in the Amhara Region, Northwest Ethiopia.

**Methods:**

A multicenter retrospective cohort study was conducted on 285 patients in the age group 15 years or older under follow-up from 1 January 2015 to 31 December 2019. Descriptive analyses were summarized using the Kaplan–Meier survival curve and the log-rank test. Then, the Cox-proportional hazard regression model was employed to estimate the hazard of death up to 5 years after they were admitted to the HF department to follow up on their treatment.

**Results:**

Out of 285 patients with HF, 93(32.6%) of the respondents were dying within 5 years of follow-up. Anemia was the common comorbid disease (30.5%), and valvular heart disease was the most common etiology (33.7%) of chronic heart failure in this study. This study showed a significant mortality difference between hospitals. HF patients with hypertension [adjusted hazard ratio (AHR): 3.5076, 95% confidence interval (CI): 1.43, 8.60], anemia (AHR: 2.85, 95% 1.61, 5.03), pneumonia (AHR: 2.02, 95% 1.20, 3.39), chronic kidney disease (2.23, CI: 1.31, 3.77), and diabetes mellitus (AHR: 2.42, 95% CI: 1.43, 4.09) were at a higher risk of death. Moreover, patients with symptoms listed in the New York Heart Association Class (III and IV), Ischemic Heart Disease and unknown etiologies, men (AHR: 2.76, 95%:1.59, 4.78), and those with a high pulse rate (AHR: 1.02, 95%:1.00, 1.04) were at a higher risk of death.

**Conclusion:**

There was a mortality difference between hospitals. This study has revealed that HF patients with anemia, diabetes mellitus, pneumonia, hypertension, chronic kidney disease, HF etiologies, severe New York Heart Association Class (III and IV), men, and high pulse rate were the main factors associated with death. Health professionals could give more attention to patients whose pulse rate is high, men, and a patient who had comorbidities in the ward.

## Introduction

Heart failure (HF) is a major health problem that affects patients and healthcare systems worldwide (1). It is the leading cause of morbidity and mortality and negatively impacts the quality of life, healthcare costs, and longevity (2). It is a pandemic, and at present, an average of 64.3 million people are living with HF worldwide (3). It is also associated with high morbidity and has a significant impact on healthcare expenditures in developed countries (4–6).

Chronic heart failure (CHF) is the final common pathway of various cardiac diseases and is characterized by high morbidity and mortality (7). While morbidity due to CHF is high in many parts of the world, the etiologies are different. The most common underlying cause of HF in high-income countries is coronary artery disease. In Sub-Saharan Africa (SSA), the predominant causes were rheumatic heart disease, hypertensive heart disease, cardiomyopathy, and Cor pulmonale (8). Low-income nations were disproportionately affected by preventable causes of HF, such as rheumatic heart disease and hypertension (9).

Patients with CHF often have multiple factors that accelerate disease progression to a greater or a lesser extent and worsen the response to treatment (10, 11). During CHF management in patients, wide ranges of comorbidities pose important challenges. Since the impact of these comorbidities and their interactions remain incompletely understood, predicting patients' clinical courses is difficult (12). It can increase morbidity and mortality, complicate the care of these patients, and affect the quality of life for patients with CHF (13, 14). HF is an increasingly common condition, and patients often experience persistent symptoms and poor quality of life, even when they are receiving the best possible treatment for their HF. Due to the high prevalence of comorbidities with HF, many coexisting medical conditions have been independently associated with the increased risk of morbidity and mortality (15). As such, optimal management of existing comorbidities in the setting of CHF is particularly important to prevent disease progression, reduce CHF hospitalizations, and improve quality of life (16).

Mortality in patients with CHF remains high, but the causes of death were not well defined. Despite the high death rate among patients with CHF, most kinds of HF can be prevented with a healthy lifestyle. Even once HF has been established, premature fatalities can be avoided by seeking medical help as soon as possible. Most patients with HF have other conditions that dominate their health experience (17). To improve outcomes for patients with HF and to ultimately save the lives of the patients, identifying the determinant factors of HF was important. Adequate studies to describe the rates of death in hospitalized patients with HF were not adequately studied in Ethiopia. Furthermore, in this specific demographic, critical factors related to in-hospital death have not been addressed in Northwest Ethiopia. However, multiple factors associated with the death of patients with CHF still need to be assessed. Thus, this study was conducted to address this issue and to identify the determinant factors of death among patients with CHF in three selected Amhara Region referral hospitals, in Northwest Ethiopia.

## Methods

### Study Area and Study Design

Our study area was purposely selected from three Referral Hospitals in the Amhara Region, namely, Debre Tabor Referral Hospital (DTRH), Felege Hiwot Specialized and Comprehensive and Specialized Hospital (FHCSH), and the University of Gondar Comprehensive and Specialized Hospital (UoGCSH), with the respective locations at 666 km, 578 km, and 725 km from Addis Ababa, the capital city of Ethiopia. DTRH is the only referral hospital in the zone and surrounding regions, serving 2.3 million people with curative and preventive health treatment. There are 91 beds available for inpatient services and 12 outpatient departments (OPDs). Patients with specific chronic conditions are referred to the hospital's specialty chronic illness clinics for follow-up (18). FHCSH is a tertiary referral and teaching hospital with 400-bed and around 15 adult OPDs that serve over 7 million people in the surrounding area (19). UoGCSH is a tertiary teaching and referral hospital in Northwest Ethiopia that has over 450 inpatient beds and provides referral health services to over 5 million people. This hospital provides a variety of services to the community, including chronic disease treatment. It has 13 distinct wards and 14 different OPDs (20). A multicenter retrospective cohort study was used.

### Duration of Study

The duration of the study was 5 years. The investigator reviewed the medical profile of each patient from his or her charts. The study period was between 1 January 2015 and 31 December 2019.

### Study Population

In this study, patients with CHF in age groups 15 years or older were selected. All patients with CHF follow-up in all Amhara Region Referral Hospitals were our target population. All randomly selected patients with CHF who took CHF treatment for a minimum of 1 month during the follow-up period in the study hospitals were included. Patients with incomplete baseline variables and patients with acute heart failure were not considered for this study.

### Source of Data and Method of Data Collection

In this study, we used a secondary source of data. The data were obtained from three selected referral hospitals in the Amhara region. The variables that we used in this study were extracted from patients' chart which contains epidemiological, laboratory, and clinical information of all patients with CHF under follow-up including a detailed HF history and socio-demographic variables. The data were collected by healthcare service providers of the CHF clinic after we had given adequate orientation for them about the way of data collection and the variables that were included in this study.

### Sample Size Determination and Sampling Procedure

The sample size estimation was determined by considering financial constraints, time constraints, and data analysis techniques. Before the actual data were collected, emphasis was made on the determination of the sample size, which mainly depends on the purpose of the study, the available resources, and the precision required. By taking the proper sample size, the degree of precision required for generalization was increased. Thus, the sample size determination formula (21) adopted for this study is as follows:


(1) $$\begin{array}{l} n_{0}=\;\frac{\frac{Z^{2}P\left(1-P\right)}{d^{2}}}{1+\frac{1}{N}\left\{\frac{Z^{2}P\left(1-P\right)}{d^{2}}-1\right\}}, \end{array}$$

where *no* is the sample size needed;

*N* is the total population size of the patients with CHF in three selected referral hospitals (*N* = 4,064);

*Z* is the upper α/2 values of standard normal distribution, and, for this study, we used a value of α = 0.05 as the significance level, which is *Z* = 1.96;

*P* is the proportion (death of patients with CHF); and

*d* is the level of precision (maximum allowable error).

The specification of *d* must be small to have good precision (21). We used the maximum allowable difference between the maximum likelihood estimate and the unknown population parameter (*d* = 0.05). We used the probability of the event, that is, 31.3% of the total patients, and this was obtained from a previous study in Ethiopia based on the University of Gondar referral hospital (16). Therefore, we used *P* = 0.313 as the probability (proportion) of death. Therefore, *no* = 306, and if $\frac{no}{N}$ > 5%, we should use $\frac{no}{1+\frac{n0-1}{N}}$, so the required sample size is *n* = 285.

The total number of patients with CHF in the study period in each hospital was *N*_*DTRH*__=_ 1,140, *N*_*UoGCSH*_ = 1,354, and *N*_*FHCSH*_ = 1,570. We used proportional allocation to select the sample from each hospital. The proportion was calculated as follows: the total number of CHF patient follow-ups on a CHF clinic at a given hospital between 1 January 2015, and 31 December 2019, multiplied by our calculated sample size (285), and then divided by the total number of patients with CHF who started CHF follow-up in the three hospitals in the study period (*N* = 4,064). The total sample size in each hospital was *n*_*DTRH*__=_ 80, *n*_*UoGCSH*_ = 95, and *n*_*FHCSH*_ = 110. A simple random sampling technique was employed to select a representative sample from each hospital.

### Study Variable

#### Response Variables

The response variable (outcome variable) for this study is the time to death of patients with CHF. In this study, the patients who had experienced death during the observation period were the event of interest. The patients who had not experienced death during the follow-up period were censored. These included patients with CHF lost to follow-up, those who were referred to other health institutions, those who were discharged with improvement, or those who stayed with admission beyond the study period.

#### Independent Variables

The predictors associated with the time to death of patients with CHF are either socio-demographic variables or clinical variables. These variables are gender, age, CHF type (left ventricular failure, right ventricular failure, and biventricular failure), hospitals (DTRH, UoGCSH, and FHCSH), etiology of HF (VHD, HHD, IHD, Cor pulmonale, dilated cardiomyopathy, and other etiologies), NYHA class (Class II, Class III, and Class IV), LVEF, residence (rural, urban), pulse rate, respiratory rate, systolic blood pressure, diastolic blood pressure, weight, and presence of atrial fibrillation, diabetes mellitus, hypertension, pneumonia, chronic kidney disease (CKD), and anemia as comorbidities.

### Data Management and Statistical Analysis

SPSS version 23.0 was used for data entering. R version 4.0.3 statistical software was used for statistical analysis. Data are described by numbers and percentages or by means and standard deviations depending on the scale of measurements. Descriptive statistics for continuous variables were summarized using mean and standard deviation. For comparison between groups, exclusively nonparametric tests were used. The survival probability among patients with CHF from the starting date to the follow-up to the event was estimated using the Kaplan–Meier survival curve. After we estimated the survival probability, the Cox proportional hazard regression model was fitted. All variables with a *P* ≤ 0.25 in the bivariable analysis were included in a multivariable analysis. The Cox-proportional hazard model assumption was checked using a formal statistical test, the GLOBAL test. In the final model, hazard ratios with 95% confidence intervals (CIs) and *P*-values (< 0.05) were used to identify statistically significant predictors and to measure the strength of association.

### Ethical Approval

Since the data collection was conducted retrospectively from patient medical record charts and patients were not directly involved in data collection, informed consent from patients was not applicable. To access patients' medical record charts, ethical clearance was obtained from the Institutional Review Board, Faculty of Natural and Computational Science, Debre Tabor University, Ethiopia, with reference number: RCS/181/2019. This study was conducted in accordance with the guidelines of Good Clinical Practice and the Principles of the Declaration of Helsinki.

## Results

The data consist of 285 congestive patients with HF who were treated under CHF follow-up in three selected hospitals, Amhara, North Western Ethiopia. We have a 100% response rate. The survival endpoint of interest is the time to death of patients with CHF. Thus, 93(32.6) patients were dying at the time of the study while the remaining 192 (67.4%) patients were censored.

Table 1 shows the result of characteristics of categorical predictor variables of patients with CHF. Out of the total 285 patients, 150 (52.6%) were female and the remaining 135 (47.4%) were male. Approximately 67 (23.5%) male and 26 (9.1%) female respondents died. Nearly two-thirds of 188 (66.0%) patients were rural residents, and 83 (29.1%) rural patients have died within 5 years. Notably, 80 (28.1%), 95 (33.3%), and 110 (38.6%) patients were considered from each hospital, DTRH, UoGCSH, and FHCSH, and 14 (4.9%), 33 (11.6%) and 46 (16.1%) patients from each hospital have died, respectively. Approximately 94(33.0%) patients had bi-ventricular HF, 106 (37.2%) patients had left ventricular failure, and the rest 85 (29.8%) patients had right ventricular failure. Notably, 66 (23.2%) patients with CHF were NYHA class II, 74 (26.0%) were class III, 145 (50.9%) were class IV, and 3 (4.5%), 12(16.2%), and 78(53.8%) patients of classes II, III, and IV, respectively, died. In addition, from the comorbidities of patients with HF, of the 70 (24.6%) patients with hypertension, 46 (67.5%) of them died, of the 75 (26.3%) patients with diabetes mellitus, 50 (66.7%) of them died, of those 62 (21.8%) with pneumonia, 43 (69.4%) died, of those 79 (27.7%) with CKD, 43 (54.4%) died, of those 87 (30.5%) with anemia, 64 (73.3%) died, and of those 55 (19.3) with AF, 41 (25.5%) of them died. Furthermore, the etiology of HF is predominantly VHD, followed by dilated cardiomyopathy and HHD. Notably, 96 (33.7%) patients had HF caused by VHD, 43 (15.1%) by HHD, 24 (8.4%) by IHD, 33 (11.6%) by cor pulmonale, 56 (19.6%) by dilated cardiomyopathy, and the remaining 33 (11.6%) of patients had HF by other etiologies. The remaining variables are described in the same way.

**Table 1:** Results of descriptive measures of categorical variables of patient's data with CHF taken at three hospitals from 2015 to 2020.

**Characteristics**	**Category**	**Number of patients (%)**	**Death**	
			**Yes (%)**	**No (%)**
Gender	Female	150 (52.6%)	26 (17.3%)	124 (82.7%)
	Male	135 (47.4%)	67 (49.6%)	68 (50.4%)
Residence	Rural	188 (66.0%)	83 (44.1%)	105 (55.9%)
	Urban	97 (34.0%)	10 (10.3%)	87 (89.7%)
Hospital	DTRH	80 (28.1%)	14 (17.5%)	66 (82.5%)
	UoGCSH	95 (33.3 %)	33 (34.7%)	62 (65.3%)
	FHCSH	110 (38.6%)	46 (41.8%)	64 (58.2%)
Patient's CHF Type	Left ventricular failure	106 (37.2%)	18 (17.0%)	88 (83.0%)
	Right ventricular failure	85 (29.8%)	27 (31.8%)	58 (68.2%)
	Biventricular failure	94 (33.0%)	48 (51.1%)	46 (48.9%)
Etiology of heart failure	VHD	96 (33.7%)	28 (29.2%)	68 (70.8%)
	HHD	43 (15.1%)	13 (30.2%)	30 (69.8%)
	IHD	24 (8.4%)	9 (37.5%)	15 (62.5%)
	Cor-pulmonale	33 (11.6%)	11 (33.3%)	22 (66.7%)
	Dilated Cardiomyopathy	56 (19.6%)	19 (33.9%)	37 (66.1%)
	Other etiologies	33 (11.6%)	13 (39.4%)	20 (60.6%)
NYHA class	Class II	66 (23.2%)	3 (4.5%)	63 (95.5%)
	Class III	74 (26.0%)	12 (16.2%)	62 (83.8%)
	Class IV	145 (50.9%)	78 (53.8%)	67 (46.2%)
Diabetes mellitus	No	210 (73.7%)	43 (20.5%)	167 (79.5%)
	Yes	75 (26.3%)	50 (66.7%)	25 (33.3%)
Hypertension	No	215 (75.4%)	47 (21.9%)	168 (78.1%)
	Yes	70 (24.6%)	46 (67.5%)	24 (34.3%)
Pneumonia	No	223 (78.2%)	50 (22.4%)	173 (77.6%)
	Yes	62 (21.8%)	43 (69.4%)	19 (30.6%)
CKD	No	206 (72.3%)	50 (24.3%)	156 (75.7%)
	Yes	79 (27.7%)	43 (54.4%)	36 (45.6%)
Anemia	No	198 (69.5%)	29 (14.6%)	169 (85.4%)
	Yes	87 (30.5%)	64 (73.6%)	23 (26.4%)
AF	No	230 (80.7%)	52 (22.6%)	178(77.4%)
	Yes	55 (19.3%)	41 (25.5%)	14 (25.5%)
Censoring status	Censored	192 (67.4%)		
	Event (death)	93 (32.6%)		

*CHF, chronic heart failure; AF, atrial fibrillation; CKD, chronic kidney disease; DTRH, Debre Tabor Referral Hospital; FHSACH, Felege Hiwot Specialized and Comprehensive Hospital; HHD, hypertensive heart disease; IHD, ischemic heart disease; NYHA, New York Heart Association; UoGSACH, University of Gondar Specialized and Comprehensive Hospital; VHD, valvular heart disease*.

Table 2 displays baseline characteristics of continuous variables, i.e., age, weight, PR, RR, SBP, DBP, and LVIF of patients with CHF. The mean values with a standard deviation of baseline age, weight, PR, and LVEF of patients were 48.2 ± 19.786 years, 56.5 ± 8.436 kg, 97.8 ± 17.436, and 51.3 ± 14.462%, respectively.

**Table 2:** Baseline characteristics of the continuous variable of patient data with CHF taken at three hospitals from 2015 to 2020.

**Variables**	**N**	**Minimum**	**Maximum**	**Mean**	**Std.dev**
Age in years	285	15	85	48.2	19.786
Weight in kg	285	35	80	56.5	8.436
LVEF in %	285	15	85	51.3	14.462
PR	285	48	140	97.8	17.436
RR	285	11	68	29.21	8.623
SBP	285	80	180	116.9	17.492
DBP	285	50	105	75.4	12.205

*CHF, chronic heart failure; DBP, diastolic blood pressure; LVEF, left ventricular ejection fraction; PR, pulse rate; RR, respiratory rate; SBP, systolic blood pressure*.

### Kaplan–Meier Estimates and Log-Rank Tests

**Figure 1:**
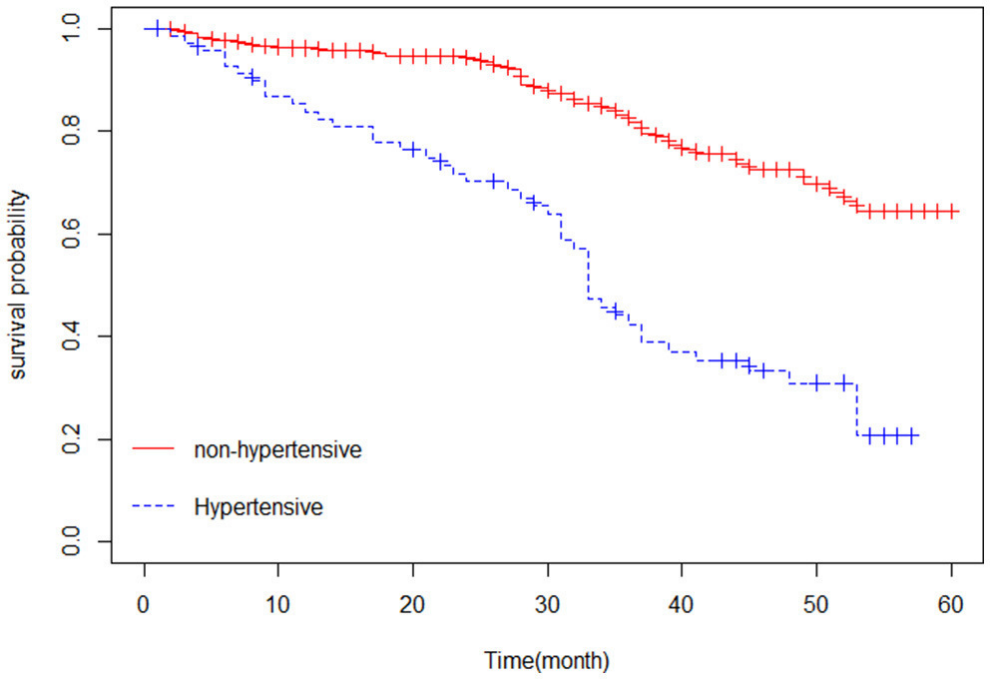
Kaplan-Meier curve of chronic heart failure patients based on hypertension status.

**Figure 2:**
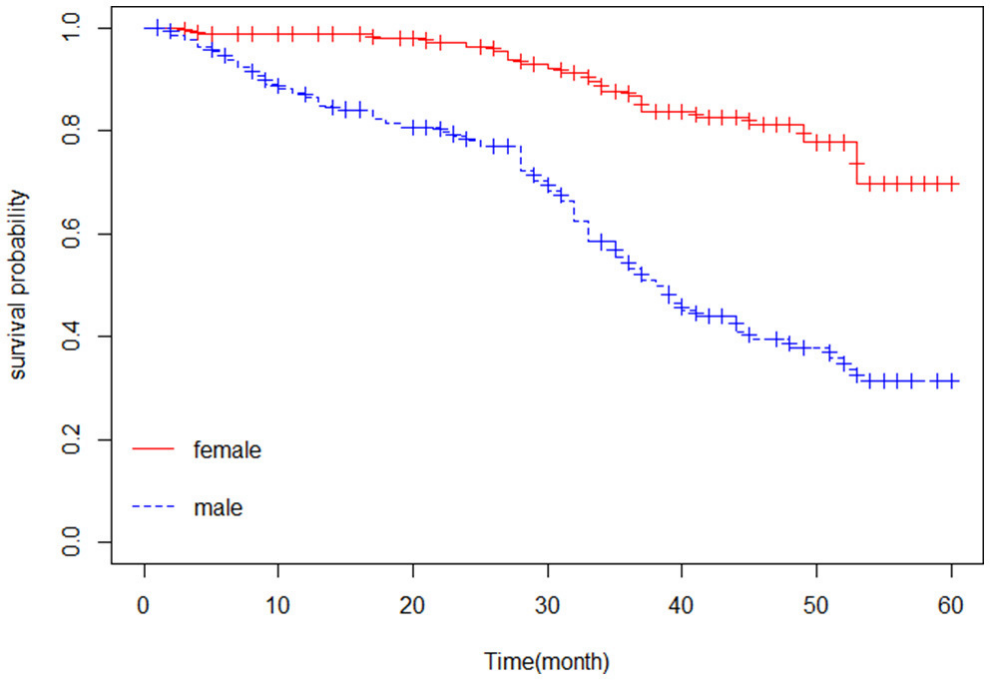
Kaplan-Meier curve of chronic heart failure patients based on gender.

The Kaplan–Meier estimator was applied to estimate the survival curves for categorical predictors. Figure 1 indicates that nonhypertensive patients have a higher probability of survival. In addition, Figure 2 shows that female patients have a higher probability of survival throughout the 5-year CHF treatment period than male patients. This means that the probability of death was higher for male patients and hypertensive patients compared with female and nonhypertensive patients (Figures 1, 2).

To check for significant differences among categories of factors, the log-rank tests were applied to all categorical variables. The null hypothesis is that there is no significant difference between the survival experiences of different groups of categorical variables.

In Table 3, the log-rank tests showed that there was a significant difference in the death rates between groups of gender, hospital, residence, CHF type, NYHA class, hypertension, pneumonia, diabetes, anemia, and CKD patients at a 5% level of significance (Table 3).

**Table 3:** Log-rank test for categorical independent variables.

**Predictors**	**Chi-square**	**Df**	***p*** **-value**
Gender	42.2	1	<0.0001
Residence	22.4	1	<0.0001
Hospital	15.6	2	<0.0001
CHF Type	17.4	2	<0.0001
NYHA class	38.5	2	<0.0001
Etiology	6.7	5	0.2
Hypertension	43.5	1	<0.0001
Diabetics	76.4	1	<0.0001
Anemia	86.9	1	<0.0001
Pneumonia	26.8	1	<0.0001
CKD	45.7	1	<0.0001
AF	73.5	1	<0.0001

### Uni-variable Cox Proportional Hazard Model

The univariable Cox proportional hazard regression models were fitted for every covariate to check covariates that affected the survival of patients with CHF before proceeding to higher models. Consequently, the candidate variables for building a multivariable Cox model are the sex of patients, the place of residence, hospital, PR, RR, SBP, DBP, NYHA class, CHF-type, etiology, and the presence of hypertension, CKD, anemia, pneumonia, and AF as comorbidity.

### Cox Proportional Hazard Assumption

The proportional hazard model assumption asserts that the hazard ratios are constant over time. This means the risk of failure must be the same, no matter how long subjects have been followed. Cox proportional hazard assumptions were checked using formal statistical tests and graphical methods. To test this assumption, GLOBAL tests were used.

Based on Table 4, the *P*-values of all covariates are >5%, indicating that the correlation between Schoenfeld residuals and survival time is not significant, which implies that all the covariates satisfy the proportionality assumption at 0.05 levels of significance and that the *P*-value of the GLOBAL test (0.0679) is not significant. This indicates that the proportional hazard (PH) assumption for the Cox model was not violated (Table 4).

**Table 4:** Proportional hazard model assumption of the Cox model for patient data with CHF at three selected hospitals from 2015 to 2020.

**Variables**	**Rho**	**Chi-square**	***P*** **-value**
SBP	−0.10	1.22	0.2699
DBP	0.12	1.74	0.1868
PR	0.21	3.65	0.0559
RR	0.09	0.71	0.3993
HTN (yes)	−0.04	0.19	0.6595
Residence(urban)	0.03	0.15	0.6969
CKD (yes)	−0.08	0.82	0.3683
NYHA class (III)	−0.10	1.37	0.2412
NYHA class (IV)	−0.02	0.01	0.9296
Etiology (VHD)	−0.14	2.40	0.1209
Etiology (HHD)	−0.13	2.17	0.1410
Etiology(IHD)	0.02	0.03	0.8640
Etiology (Cor-Pulmonale)	−0.08	0.61	0.4348
Etiology (dilated cardiomyopathy)	0.05	0.26	0.6093
Gender(yes)	−0.01	0.01	0.9585
Pneumonia (yes)	−0.09	0.87	0.3508
Anemia (yes)	−0.16	2.98	0.0841
AF (yes)	−0.10	1.24	0.2647
CHF type (right ventricular)	0.19	3.74	0.0532
CHF type (biventricular)	0.10	1.31	0.2520
GLOBAL	NA	30.12	0.0679

### Cox PH Model

All the parameter estimates were estimated by considering the other predictors. Notably, 95% of CIs for the hazard ratios of the statistically significant risk factors do not include one (the null value). In contrast, the 95% CIs for the nonsignificant risk factors include the null value. The results of the Cox proportional hazard model are presented in Table 5.

**Table 5:** Univariable and multivariable Cox regression analyses of predictors of mortality among patients with CHF in three selected Amhara Region Referral Hospitals from 2015 to 2020.

**Parameters**	**CHR (95% CI)**	**AHR (95% CI)**	***p*** **-value**
Gender (ref.=Female)	1	1	
Male	4.02 (2.55, 6.34)	2.76 (1.59,4.78)	0.001^**^
Residence (ref= Rural)	1	1	
Urban	0.23 (0.12, 0.45)	0.49 (0.23,1.03)	0.062
Hospital (ref= DTRH)	1	1	
UoGCSH	2.62 (1.40, 4.92)	2.58 (1.25,5.29)	0.009^**^
FHCSH	3.15 (1.73, 5.74)	3.64 (1.71,7.76)	0.001^**^
Hypertension (ref.=No)	1	1
Yes	3.63 (2.42, 5.46)	3.51 (1.43,8.60)	0.006^**^
CKD (ref.=No)	1	1
Yes	3.78 (2.49, 5.71)	2.23 (1.31,3.77)	0.003^**^
Pneumonia (ref.=No)	1	1
Yes	3.6 (2.40, 5.43)	2.02 (1.20,3.39)	0.008^**^
AF(ref.=No)	1	1
Yes	5.1 (3.4, 7.77)	1.10 (0.63,1.91)	0.723
Anemia (ref.=No)	1	1
Yes	6.27 (4.042, 9.752)	2.85 (1.61,5.03)	0.001^**^
DM (ref.=No)	1	1
Yes	5.37 (3.54, 8.15)	2.42(1.43,4.09)	0.001^**^
NYHA Class (ref. = Class II)			
Class III	5.03 (1.42, 17.86)	6.93 (1.92,24.99)	0.003 ^**^
Class IV	12.93 (4.08, 41.01)	8.08 (2.05,31.85)	0.003^**^
Etiology(ref.=VHD)	1	1	
HHD	0.98 (0.51,1.89)	1.26 (0.59,2.66)	0.539
IHD	1.38 (0.65, 2.93)	2.64 (1.13,6.15)	0.024 ^**^
Cor-Pulmonale	1.32 (0.66, 2.65)	0.51 (0.23,1.14)	0.103
Dilated cardiomyopathy	1.11 (0.62,1.99)	0.97 (0.50,1.89)	0.933
Other etiology's (unknown)	2.19(1.13,4.2)	0.32(0.13,0.78)	0.012^**^
CHF-type (ref.= Left ventricular)	1	1	
Right ventricular	1.87 (1.03, 3.40)	1.39 (0.70,2.75)	0.345
Biventricular	2.98 (1.73, 5.13)	0.88 (0.46,1.69)	0.708
PR	1.05 (1.04, 1.06)	1.02 (1.00,1.04)	0.015^**^
RR	1.07 (1.049, 1.082)	1.02 (1.00,1.04)	0.172
Blood pressure			
SBP	1.02 (1.01, 1.03)	0.99 (0.95,1.02)	0.498
DBP	1.03 (1.01, 1.04)	0.99 (0.95,1.05)	0.899

*Ref, reference category;*

** *indicates the significance covariates at a 5% level of significance*.

Based on Table 5, sex, hospital, hypertension, DM, anemia, CKD, pneumonia, NYHA class, etiology, and pulse rate were significant factors that increased the risk of death among patients with CHF. We observed that the hazard of death among male patients with CHF was 2.8-fold [adjusted hazard ratio (AHR): 2.76, 95% CI: 1.59, 4.78, *P* = 0.001)] higher than that among female patients. The hazard of death among patients with CHF followed up by FHCSH was 3.6-fold (AHR: 3.64, 95% CI: 1.71, 7.76, *P* = 0.001) higher and among patients with CHF followed up by UoGCSH was 2.6-fold [(AHR: HR = 2.58, 95% CI: 1.25, 5.29, *P* = 0.009] higher compared with patients with CHF followed up by their treatment in DTRH. The hazard of death of CHF patients with hypertension was 3.5-fold (AHR: 3.51, 95% CI: 1.43, 8.60, *P* = 0.006) higher than in nonhypertensive patients with CHF. The hazard of death among CHF patients with CKD was 2.2-fold higher as compared with those without CKD (AHR: 2.23, 95% CI: 1.31, 3.77, *P* = 0.003). Additionally, the hazard of death among patients with CHF who had pneumonia was 2-fold [AHR: 2.02, 95% CI: 1.20, 3.39, *P* = 0.008)] higher than in patients who did not have pneumonia as comorbidity. The hazard of death among anemic patients with CHF was 2.8-fold [AHR: 2.85, 95% CI: 1.61, 5.03, *P* = 0.0003)] higher compared with nonanemic patients. The hazard of death among CHF patients with diabetes was 2.4-fold [AHR: 2.42, 95% CI: 1.43, 4.09, *P* = 0.001)] higher compared with nondiabetic patients. The hazard of death among NYHA Class IV patients with CHF was 8-fold (AHR: 8.08, 95% CI: 2.05, 31.85, *P* = 0.003) higher and among NYHA Class III patients was 7-fold ((AHR: = 6.93, 95% CI: 1.92, 24.99, *P* = 0.003) higher compared with NYHA Class II patients. This means that there was an increasing risk of death for NYHA Class IV patients and NYHA Class III when compared with NYHA Class II patients.

Furthermore, the hazard of death among patients with CHF caused by IHD was 2.6-fold (AHR: 2.64, 95% CI: 1.13, 6.15, *P* = 0.024) higher and those caused by other etiologies were 0.3-fold (AHR: 0.32, 95% CI: 0.13, 0.78, *P* = 0.012) higher compared with patients caused by VHD. Lastly, a higher baseline heart rate was a significant predictor of mortality (HR = 1.02, 95% CI: 1.00, 1.04, *P* = 0.015). When PR is increased by one unit, the expected hazards of death of the patient are increased by 2% (Table 5).

## Discussion

This study examines the effect of comorbidities and other factors on the survival time of patients with CHF. It also demonstrated that a higher heart rate was associated with adverse outcomes such as a high risk of mortality among patients with CHF. In the multivariable Cox proportional hazard model, sex, hypertension, CKD, pneumonia, diabetes mellitus, anemia, hospital, NYHA class, etiology/cause of HF, and pulse rate are significantly associated with the hazard of death.

In this study, we found that male patients had a higher risk of mortality compared with female patients. This result supported by most of the studies (16, 22, 23) showed that female patients had a slightly higher survival probability than male patients. In line with other studies (24–26), these studies indicate that the male gender had a high risk of mortality. However, this study contradicts two earlier studies (27), one which showed no significant differences in mortality among the gender of patients with HF and the other (28) which showed that female patients had a higher hazard of mortality.

Anemia was the common comorbid disease (30.5%) in this study. The risk of death among anemic patients with CHF was 2.8-fold higher as compared with their nonanemic counterparts. Similar to previous studies (11, 28–31), we found that CHF patients with anemia had a high risk of mortality. The risk of death among CHF patients with diabetes mellitus was 2.8-fold higher compared with patients with nondiabetic mellitus. These results are in agreement with the previous findings (11, 22, 24, 28, 31–37). Patients with both diabetes mellitus and HF are particularly at an elevated risk of death compared with nondiabetic patients. This study contradicts the previous study (27), which showed that DM has no significant death effect on patients with HF.

Another finding of this study revealed that the death rate among patients with CHF with advanced class [NYHA class (IV) and NYHA class (III)] was higher compared with patients with NYHA class (II). This finding was highly supported by previous studies (24, 38), which found that a greater NYHA class worsened the quality of life of patients with HF. This finding contradicted the previous study (27), which showed that NYHA has no significant effect on the death of patients with HF.

This study reveals that patients with CHF presenting with hypertension were at higher risk of mortality. This finding was in line with the previous findings (28, 29, 35, 39, 40), which showed that hypertension had a positive significant effect on the prevalence of CHF. Similarly, mortality due to CHF was significantly higher in patients with pneumonia and CKD, which is in line with the studies of Jobs et al. (41) and van Deursen et al. and Senni et al., (31, 40) respectively.

Further findings of this study demonstrated that VHD was the most common etiology (33.7%) in this study. However, patients with CHF caused by IHD were at a higher risk of death compared with patients with CHF caused by VHD. This finding is in agreement with the previous Ethiopian studies (42). A significant positive, linear relationship was observed for both baselines and serially measured pulse rates with all-cause mortality (43). This finding is not in line with the previous study (30) that found no significant relationship between CHF and any of the etiologies of CHF (ischemic heart disease, dilated cardiomyopathy, hypertensive heart disease, valvular heart disease, and other etiologies) except cor pulmonale. This contradicted another study (44), which found that death outcomes were similar across etiological categories.

A higher baseline heart rate was known to be a significant predictor of death. This study was in line with the studies(43, 45), showing that a higher heart rate/pulse rate was associated with a high hazard of mortality. Both baselines and serially recorded pulse rates were found to have a significant positive, linear relationship with all-cause death (43). A higher baseline heart rate was a significant predictor of mortality, and reducing the heart rate improves prognosis in patients with HF (46). The hazard of death for the patients who were treated at FHCSH and UoGCSH was higher compared with patients with CHF following their treatment follow-up at DTRH. This was due to patients with severe HF being treated at one of the comparative and specialized hospitals.

### Strengths and Limitations

This study was performed in a multicenter setting, which can enhance the generalizability of the data to the entire population. In addition, this study has provided a real insight into the current clinical pattern among hospitalized patients with CHF in Northwest Ethiopia. However, at the same time, there were certain limitations in this study. First, due to the retrospective nature, the data obtained might be affected by the documentation culture of the hospital and the healthcare providers. Second, potentially relevant variables such as body mass index, alcoholism, marital status, education level, and smoking status were not included.

## Conclusion

In this study, we aimed to examine the time to death and its determinant factors among patients with CHF in Northwest Ethiopia. According to the finding, men, hypertension, CKD, pneumonia, diabetes mellitus, and anemia were positive significant factors of death compared with their counter groups. There is a mortality difference between hospitals. The highest heart rate was associated with a high risk of mortality among patients with CHF. Health professionals could provide more attention to patients with CHF whose pulse rate is high and to patients with comorbidities of hypertension, coronary kidney disease, pneumonia, diabetes mellitus, and anemia in the ward. Finally, to show the association factor between longitudinal and survival analyses, a future extension of this study—a joint model of longitudinal measure pulse rate and time to death—is recommended.

## Data Availability Statement

The original contributions presented in the study are included in the article/supplementary materials, further inquiries can be directed to the corresponding author.

## Ethics Statement

The studies involving human participants were reviewed and approved by to access patient's medical record charts, Ethical Clearance was obtained from Debre Tabor University Institutional Review Board of Faculty of Natural and Computational Science, Ethiopia, with reference number: RCS/181/2019. Written informed consent from the participants' legal guardian/next of kin was not required to participate in this study in accordance with the national legislation and the institutional requirements.

## Author Contributions

YM contributed to the write-up, development of the proposal, data collection format, data entry, data analysis, and write-up of the manuscript. MM, AB, and SF participated in the design and data analysis, critically read the manuscript, and edited the manuscript. All authors have read and approved the manuscript.

## Conflict of Interest

The authors declare that the research was conducted in the absence of any commercial or financial relationships that could be construed as a potential conflict of interest.

## Publisher's Note

All claims expressed in this article are solely those of the authors and do not necessarily represent those of their affiliated organizations, or those of the publisher, the editors and the reviewers. Any product that may be evaluated in this article, or claim that may be made by its manufacturer, is not guaranteed or endorsed by the publisher.

## Abbreviations

AF: atrial fibrillation
AHR: adjusted hazard ratio
CHF: chronic heart failure
CHR: crude hazard ratio
CI: confidence interval
CKD: chronic kidney disease
BVF: bi-ventricular failure
DTRH: Debre Tabor Referral Hospital
DBP: diastolic blood pressure
FHSACH: Felege Hiwot Specialized and Comprehensive Hospital
HHD: hypertensive heart disease
IHD: ischemic heart disease
K–M: Kaplan–Meier
LVEF: left ventricular ejection fraction
LVF: left ventricular failure
NYHA: New York Heart Association
OR: odds ratio
PH: proportional hazard
PR: pulse rate
RHD: rheumatic heart disease
RVF: right ventricular failure
RR: reparatory rate
SBP: systolic blood pressure
SD: standard deviation
SSA: Sub-Saharan Africa
UoGSACH: University of Gondar Specialized and Comprehensive Hospital
VHD: valvular heart disease
WHO: World Health Organization.
